# Suicidal behaviour among terminally ill cancer patients in India

**DOI:** 10.4103/0019-5545.55950

**Published:** 2005

**Authors:** K.S. Latha, S.M. Bhat

**Affiliations:** * Associate Professor Department of Psychiatry, Kasturba Hospital, Manipal 576119, Udupi; ** Professor Department of Psychiatry, Kasturba Hospital, Manipal 576119, Udupi

**Keywords:** Terminal cancer, suicidal ideation, desire for death

## Abstract

**Background:**

Passive suicidal thoughts are relatively common in patients with terminal cancer. There is a need for more information about the factors that influence these patients to desire death.

**Aim:**

To examine the prevalence of suicidal ideation among terminally ill cancer patients.

**Methods:**

Fifty-four terminally ill inpatients (27 men and 27 women) from the palliative care unit of the Oncology department of Kasturba Hospital, Manipal were evaluated on various rating scales for depression, hopelessness and suicidal ideation, and the correlation of suicidal ideation with medical symptoms such as pain, as well as awareness of the diagnosis and understanding of the illness.

**Results:**

Most patients (79.7%) denied having suicidal thoughts or wishing for an early death; only 9.2% had severe suicidal ideation. Two patients (3.8%) with severe suicidal ideation had a past history of major depression. Factors such as the presence of pain, awareness of the diagnosis and understanding of the illness contributed to depressive mood states.

**Conclusion:**

Suicidal ideation and a desire for death appear to be linked exclusively to the presence of a mental disorder. In addition, poor pain control, and awareness of the diagnosis may also contribute to suicidal ideation.

## INTRODUCTION

Suicide rates tend to correlate with the degree of acceptance of suicidal behaviour in particular cultures. In India, for instance, religion forbids death by suicide and it is believed that one who commits suicide will not attain *moksha* (self-liberation); it is considered a sin. However, the epics mention the ending of one's life as justified under certain circumstances such as when a person is suffering from an incurable disease or intolerable pain. In ancient times, people who were too old to carry out their religious duties used to terminate their lives voluntarily by drowning, starvation, self-cremation *(samadhi),* etc.^1^–^3^ Social deterrents have also played an important role in preventing a person from committing suicide; for example, family attachments and commitments, and social stigma played a buffering role. However, a change in the values and belief systems of individuals as a result of education and exposure to the mass media has brought about a change in the attitude towards suicide. The actual incidence of suicide in these patients is probably underestimated, as there may be reluctance to report such deaths in these circumstances.^4^

Some studies suggest that while relatively few cancer patients commit suicide, they are at increased risk for suicide.^5^–^7^ Passive suicidal thoughts are relatively common among them. The relationships among suicidal thoughts and the desire for hastened death, requests for physician-assisted suicide, and/ or euthanasia are complex and poorly understood.^8^ The understanding of the concept of euthanasia and physician-assisted suicide is in a primal stage in developing countries.

In a study of 44 terminally ill patients, 77.3% had never wished for death to come early. Three patients had been suicidal and 7 others had desired early death. All 10 patients who had desired death were found to be suffering from clinical depressive illness.^9^

Another study examined 200 terminally ill inpatients by a semi-structured interview. Occasional wishes for death to come soon were reported by 44.5% of patients; 8.5% of the patients acknowledged a serious and pervasive desire to die. The desire for death was correlated with ratings of pain and low family support but most significantly with measure of depression. The prevalence of diagnosed depressive syndrome was 58.8% among patients with a desire to die and 7.7% among patients without such a desire.^10^

There is a paucity of data on the incidence of suicidal ideation in terminally ill cancer patients. We investigated the occurrence of suicidal ideation/desire for death in this group to determine its association with the putative risk factors of depression, pain, awareness of the diagnosis, and the course and outcome of the illness.

There is also a need for more information about the factors that influence terminally ill cancer patients to desire death, especially factors that might be remediable with medication and psychosocial interventions.^11^–^13^

## METHODS

Seventy-six patients with a primary medical diagnosis of terminal cancer (life expectancy ≤6 months), were recruited from the palliative care unit of Kasturba Hospital, Manipal, India. Of these 76, 22 (28.9%) had to be excluded—7 (9.2%) had cognitive impairment, 6 (7.9%) had inability to communicate including impaired sensorium, 5 (6.6%) were physically weak or sick, and 4 (5.3%) died. Thus, the final group interviewed comprised 54 patients (27 men and 27 women). Diagnostic classification or treatment regimen was not a determining factor for selection. The study was approved by the ethics review committee of the hospital and all patients gave their consent to participate. Each patient underwent a semi-structured diagnostic interview leading to a DSM-IV diagnosis. In all cases the interview was conducted by the first author (KSL) who had been trained in the protocol. The psychological status of the patients was assessed through a series of psychiatric rating scales.

### The Beck Depression Inventory (BDI), 1974

This is a 21-item self-report inventory. Each item consists of four alternative statements that represent gradations of a given symptom rated in severity from 0 to 3. The instrument was either self-administered by the patient or read aloud by the examiner and rated. Symptoms such as anorexia, weight loss, insomnia, reduced energy and fatigue were taken as physical items which could be a part of any medical condition and hence excluded from the total score.

### Hopelessness Scale (HS)^14^

This consists of 20 True–False statements to assess the extent of pessimism. Each of the 20 items score 1 or 0. The total score is the sum of the individual item scores. The possible range of scores is from 0 to 20. This scale was also either self-administered by the patient or read aloud by the examiner and rated.

### The Scale for Suicide Ideation (SIS)^15^

This scale quantifies the severity of current suicidal thoughts and wishes. It includes 19 items, each with 3 choices—0 (least severe), 1 (severe) and 2 (most severe). The total score is computed by summing up the individual item ratings; it can range from 0 to 38. The items quantify the frequency and duration of suicidal thoughts as well as the patient's attitude towards them. Subjective feelings of control regarding suicidal ideation are also assessed. The scale was completed by the examiner in a semi-structured interview with the patient.

*Assessment of the impact of medical factor (e.g. the presence of pain) on mental status:* The intensity of pain was measured by the verbal rating scale comprising three descriptors-‘no pain’ or ‘least possible pain’, ‘pain controlled’, and ‘worst possible pain’.

*Awareness of the diagnosis:* The following details were collected in a semi-structured interview.

- Patient's awareness of the diagnosis and understanding of the illness, details of its meaning, prognosis, course and outcome.
- What are the patient and the family being told?
- What and how much are they absorbing?

Awareness of the diagnosis and understanding of the illness were graded as ‘present’, ‘partial/denied’, and ‘absent’.

In the statistical analysis, unadjusted responses from the initial interview were used to determine the overall prevalence of suicidal ideation/desire-for-death phenomenon. Demo-graphic, psychiatric and medical correlates of suicidal ideation/desire for death were then examined. Descriptive statistics were generated for all the variables. Mean scores were computed for depression, hopelessness and suicidal ideation. ANOVA was done to compare the impact of medical variables such as pain, awareness of the diagnosis and the meaning of illness on the variables of depression, hopelessness and suicidal ideation on different patients.

## RESULTS

The sociodemographic profile of the study group is given in Table 1. The mean age of men was 51.3 years (SD±12.5, range: 22–77) and of women 45.1 years (SD±10.8, range: 22–67). In most patients (44.4%), the gastrointestinal tract was the primary site of tumour. The majority of patients were from nuclear families (66.7%), were married (77.8%) and had not attended school (34%). In most cases, the patient was the main bread-winner (29.6%) followed by the spouse (27.8%).

**Table 1:** Demographic profile of the study group

Variable	%	Variable	%
Primary tumour sites		Marital status	
Gastrointestinal tract	44.4	Married	77.8
Genitourinary tract	33.3	Never married	11.1
Lungs	9.3	Widowed	7.4
Female breast	3.7	Divorced	3.7
Haematological malignancies	3.7	Educational level	
		Never attended school	34.0
Solid tumours	5.6	<High school education	29.0
Family structure		High school	29.6
Nuclear families	66.7	College	7.4
Joint/extended families	31.5	Employment status	
		Employed	42.6
Living alone	1.8	Unemployed	22.2
		Housewives	29.6
		Retired	5.6

The duration of illness was 1–6 months in 61% of the patients; 6 months–1 year in 20.4%; and >1 year in the remaining. Of the men, 74.1% had a history of alcohol intake; about 45% of these men had dependence and the rest drank occasionally. The distribution of illness-related variables among the study group is given in Table 2.

**Table 2:** Distribution of illness-related variables among terminally ill cancer patients (*n*=54)

Variable	*n*	%
Presence of pain	28	51.8
Awareness of the diagnosis	26	48.1
Aware of the meaning of illness	19	35.2
H/o of alcohol use	20 (*n*=27)	74.1
Alcohol dependence	9 (*n*=20)	45.0
H/o of physical illness	10	18.5
Past h/o cancer	6	11.1
Past h/o of psychiatric illness	2	3.7
Past h/o suicide attempt	—	—
Family h/o psychiatric illness	20	37.0
1 relative affected	13	65.0
2 or more relatives affected	7	35.0
Nature of psychiatric illness		
Alcohol dependence*	9	45.0
Mood disorders	6	30.0
Completed suicide†	3	15.0
Other	2	10.0

* Two patients with alcohol dependence also had an affective disorder

† Three completed suicides had a diagnosis of an affective disorder

Table 3 gives the distribution of BDI, HS and SIS scores across the medical variables mentioned above. In this study, the mean depression score of patients who knew the meaning of their illness was lower than in those who were ignorant of the meaning (19.1 vs. 22.1). ANOVA did not show any statistical significance. The results of the mean HS and SIS scores were also not significant.

**Table 3:** Distribution of depression, hopelessness and suicidal ideation scores across the medical variables of pain, awareness of the diagnosis and meaning of the illness

Variable	*n* (%)		BDI Mean (SD)	HS Mean (SD)	SIS Mean (SD)
Awareness of the diagnosis					
Present	26	(48.1)	21.7 (8.7)	12.8 (5.2)	4.7 (6.9)
Partial	3	(5.6)	23.7 (6.1)	8.7 (9.6)	8.0 (0)
Absent	23	(42.6)	20.8 (7.9)	8.3 (5.4)	1.8 (0.70)
Denied	2	(3.7)	4.00 (5.6)	4.00 (5.6)	0 (0.0)
			df: 3	df: 3	df: 3
			f: 3.022	f: 3.521	f: 1.184
			p<0.05	p<0.05	p=0.325 NS
Meaning of the illness				
Present	19	(35.2)	19.1 (9.0)	12.1 (5.13)	2.4 (5.7)
Partial	3	(5.6)	17.0 (2.64)	10.0 (1.0)	3.0 (0.0)
Absent	32	(59.2)	22.1 (8.2)	9.3 (6.9)	4.06 (6.04)
			df: 2	df: 2	df: 2
			f: 1.004	f: 1.328	f: 0.347
			p=0.374 NS	p=0.274 NS	p=0.708 NS
Presence of pain				
Present	28	(51.8)	25.2 (7.6)	12.8 (5.7)	5.8 (6.6)
Controlled	5	(9.3)	17.8 (5.4)	10.4 (3.4)	0 (0.0)
Absent	21	(38.9)	15.5 (7.5)	6.9 (5.3)	0.64 (10.6)
		df: 2	df: 2	df: 2
		f: 10.87	f: 0.117	f: 2.57
		p<0.001	p<0.001	p<0.05

The mean depression score in patients who were aware of the diagnosis was 21.7, whereas it was 20.8 in those who were not aware of the diagnosis. The degree of awareness of the diagnosis was significantly correlated with development of depression as well as hopelessness. The differences in the mean score of SIS were not statistically significant.

In this study, 51.8% of the patients had pain. There were differences in the mean BDI, HS and SIS scores between the three groups on the variable of pain; these were statistically significant. The mean depression and hopelessness scores were higher in those patients in whom the pain was poorly controlled.

It is evident from Table 4 that suicidal ideation was an uncommon occurrence. Forty-three patients (79.7%) denied having suicidal ideation and 10 (18.5%) had moderate-to-severe suicidal ideation; 4 of the 10 patients (40%) expressed repeated suicidal thoughts whereas others had fleeting thoughts of suicide. Nine (16.7%) severely depressed patients had a BDI score >30; 5 (9.2%) had severe and 4 (7.4%) moderate hopelessness. Two of the 9 patients who were severely depressed had a past history of major depression and 1 was treated. One severely depressed woman repeatedly pleaded for termination of her life.

**Table 4:** Distribution of occurrence of suicidal ideation

SIS (score range)	SI	*n*	%
0–3	Nil/denied	43	79.7
4–8	Mild	1	1.9
9–17	Moderate	5	9.2
≥18	Severe	5	9.2
Total		54	100.0

SIS: Suicide Ideation Scale; SI: suicidal ideation

## DISCUSSION

This study provides information about the occurrence of suicidal ideation in terminally ill cancer patients. A patient with terminal cancer goes through various emotional stages—denial, anger, bargaining, depression and finally acceptance. In the stage of depression one might experience hopelessness and fleeting suicidal thoughts. However, these reactions may be dependent on such physical and psychological variables as pain, awareness of the diagnosis, understanding of the meaning of cancer in terms of its course, prognosis and outcome, social support, previous coping style and financial standing. The site of illness and disability as a result of the illness might affect the mood of the patient.

The robust association of suicidal ideation with current depressive symptoms leads to the hypothesis that suicidal ideation/desire for death may occur with greater frequency among patients who have increased vulnerability to depression as might be reflected in their psychiatric history. This hypothesis received partial support as only 2 of the 9 severely depressed patients had a past history of depressive episodes and others expressing a death wish had no past history of depressive episodes.

Patients with terminal cancer develop suicidal ideation/ desire for death in different ways, and their experience helps to frame the terms of reference in planning various psychological interventions as well as in the euthanasia debate. In this study, a 60-year-old married woman with severe depression and hopelessness expressed repeated suicidal thoughts and pleaded for mercy killing. She had a past history of breast cancer and had undergone mastectomy. She later developed cancer of the oesophagus with metastasis. She had difficulty in swallowing, talked in whispers and wept incessantly whenever the examiner visited her. Her husband was always by her side and appeared caring and supportive. She reported that they were childless and no one would be affected by her death. She died 1 week later in the hospital. Two other patients (a male and a female) in their mid-forties had severe suicidal ideation. They had a past history of major depression and one of them was on treatment. Two other men in this group reported lack of financial support as a reason for suicidal ideation. Being the main bread-winners, their finances were affected, and they had unfulfilled commitments such as the marriage of a daughter, sick children to care for and no one else to take the responsibility of their wives and children after their death.

Understanding and managing the problem of pain in cancer patients is of special importance. Pain is significantly associated with high levels of depression and anxiety. Chronic pain may be depression equivalent and may cause psychiatric syndrome. In this study about 51.8% of patients complained of pain which was distressing and 16.7% had a BDI score above 30. Depression, hopelessness and poorly controlled pain may be risk factors for suicide in patients with cancer.^8^

It is interesting to note that about 79.7% of patients denied having suicidal ideation. This may be due to the fact that while many may harbour suicidal thoughts at some time or other in the course of their illness, they refrain from expressing suicidal ideation for fear of criticism from the family and treating team as death by suicide is stigmatized. Many were unaware of the concept of euthanasia and therefore did not ask about mercy killing. There also might have been some protective factors in the family and community at large due to which patients refrained from expressing suicidal ideation/death wishes. Belief in the theory of *karma,* fate and destiny may be yet other reasons why, despite the worst life situations, people do not verbalize their death wishes. It should also be noted that all the patients had satisfactory social support networks, which might have facilitated coping with their crisis.

Patients who are suicidal require careful assessment to determine the underlying cause—whether it is a depressive disorder or an expression of the patient's desire to have ultimate control over the symptoms.^16^ Prompt identification and treatment of major depression is decisive in lowering the risk of suicide. It is important to assess the underlying reasons for hopelessness and death wishes. These might be related to poor symptom management, fear of a painful death or feelings of abandonment.^17^ A supportive therapeutic relationship should be maintained, which conveys the attitude that much can be done to alleviate emotional and physical pain. A crisis intervention-oriented psychotherapeutic approach that mobilizes as much of the patient's support system as possible should be initiated.
